# Selection of the Strain *Lactobacillus acidophilus* ATCC 43121 and Its Application to Brewers' Spent Grain Conversion into Lactic Acid

**DOI:** 10.1155/2015/240231

**Published:** 2015-11-10

**Authors:** Rossana Liguori, Carlos Ricardo Soccol, Luciana Porto de Souza Vandenberghe, Adenise Lorenci Woiciechowski, Elena Ionata, Loredana Marcolongo, Vincenza Faraco

**Affiliations:** ^1^Department of Chemical Sciences, University of Naples “Federico II”, Complesso Universitario Monte S. Angelo, Via Cintia 4, 80126 Naples, Italy; ^2^Department of Bioprocess Engineering and Biotechnology, Federal University of Paraná, Coronel Francisco H. dos Santos Avenue 210, 81531-990 Curitiba, PR, Brazil; ^3^Institute of Agro-Environmental and Forest Biology (IBAF), UOS Napoli, National Council of Research, Via Pietro Castellino 111, 80131 Naples, Italy

## Abstract

Six *Lactobacillus *strains were analyzed to select a bacterium for conversion of brewers' spent grain (BSG) into lactic acid. Among the investigated strains, *L. acidophilus* ATCC 43121 showed the highest yield of lactic acid production (16.1 g/L after 48 hours) when grown in a synthetic medium. It was then analyzed for its ability to grow on the hydrolysates obtained from BSG after acid-alkaline (AAT) or aqueous ammonia soaking (AAS) pretreatment. The lactic acid production by *L. acidophilus* ATCC 43121 through fermentation of the hydrolysate from AAS treated BSG was 96% higher than that from the AAT treated one, although similar yields of lactic acid per consumed glucose were achieved due to a higher (46%) glucose consumption by *L. acidophilus* ATCC 43121 in the AAS BSG hydrolysate. It is worth noting that adding yeast extract to the BSG hydrolysates increased both the yield of lactic acid per substrate consumed and the volumetric productivity. The best results were obtained by fermentation of AAS BSG hydrolysate supplemented by yeast extract, in which the strain produced 22.16 g/L of lactic acid (yield of 0.61 g/g), 27% higher than the value (17.49 g/L) obtained in the absence of a nitrogen source.

## 1. Introduction

Bioconversion of lignocellulosic residual biomass can make a significant contribution to the production of organic biochemicals [1]. Huge amounts of lignocellulosic wastes are produced yearly all around the world. They include agricultural residues, food farming wastes, green-grocer's wastes, tree pruning residues, and the organic and paper fractions of urban solid wastes. A wide range of high added value products, such as biofuels, organic acids, biopolymers, bioelectricity, and molecules, for the food and pharmaceutical industries [1] can be obtained by upgrading solid wastes through biotechnological processes.

One of the most important organic compounds is lactic acid, recognized as a GRAS (generally recognized as safe) compound by the US FDA (Food and Drug Administration), with many applications in food, cosmetics, and pharmaceutical and chemical industries [2]. Moreover, it has received a great deal of attention as a feedstock monomer for the production of PLA (polylactic acid), used as a biodegradable commodity plastic.

Microbial production of lactic acid from lignocellulosic wastes necessitates the pretreatment of lignocellulosic biomass to remove the barrier of lignin and expose the polysaccharides on the plant cell wall, the enzymatic saccharification of the polysaccharides with a (hemi)cellulolytic enzyme cocktail, and the fermentation of the resulting sugars with lactic acid producing microorganisms [3].

Among the lignocellulosic wastes, BSG is considered as a valuable low-cost feedstock with considerable attractiveness for energy production. BSG represents the major by-product of the brewing industry. It accounts for about 85% of the total residues generated after the mashing and lautering processes [4] and is available in large quantities throughout the year. The chemical composition of BSG varies according to several factors such as the barley variety, the harvest time, and the malting and mashing conditions. BSG, as with any other lignocellulosic waste, is susceptible to transformation into a variety of different value-added products.

In this paper, six lactic acid bacteria (LAB) were compared for their ability to produce lactic acid in a synthetic growth medium. The most productive strain was investigated for its ability to produce lactic acid from the sugar mixture obtained by enzymatic hydrolysis of pretreated BSGs from two different geographical areas. The hydrolysates obtained by enzymatic hydrolysis of BSGs after two types of pretreatment, aqueous ammonia soaking and acid-alkaline pretreatment, were evaluated as substrate for the growth of the selected bacterial strain and the lactic acid production.

## 2. Materials and Methods

### 2.1. Chemical Pretreatments of Brewers' Spent Grains and Enzymatic Hydrolysis of Pretreated Materials

The brewers' spent grains provided by the brewery Bier Hoff Curitiba-PR (Brazil) (BSG 1) were subjected to acid-alkaline treatment (AAT). This consisted of a first treatment with 1.25% (v/v) H_2_SO_4_ in 1 : 8 (w/w) ratio at 120°C for 17 minutes [5]. The solid residue was washed with water until the pH was neutral, dried overnight at 50 ± 5°C, and mixed with 2% (v/v) NaOH in a 1 : 20 (w/w) ratio at 120°C for 90 minutes [6]. The cellulose pulp was washed with water until the pH was neutral, and it was dried overnight at 50 ± 5°C.

The brewer spent grains provided by the microbrewery Maneba (Striano, Naples, Italy) (BSG 2) were subjected to an aqueous ammonia soaking (AAS) treatment on a lab-scale as described by Maurelli et al. [7]. The biomass milled to a fine powder was suspended in 5% (v/v) aqueous ammonium hydroxide solution at a solid to liquid ratio of 1 : 10 and incubated at 70°C for 22 hours in screw-capped 25 mL bottles to reduce the evaporation. The solid residue, recovered by centrifugation at 8000 ×g, was extensively washed with water until reaching neutral pH and dried overnight at 50 ± 5°C.

The saccharification experiments were carried out in a total volume of 50 mL containing 50 mM sodium citrate buffer pH 5.5 plus enzyme cocktail (2.24% (v/v) cellulase and 1% (v/v) *β*-glucosidase, Novozymes) at 45°C, 60 ×g for 72 hours. To reach a comparable cellulose content in the pretreated lignocellulosic substrates subjected to enzymatic hydrolysis, the pretreated BSG samples, AAT BSG 1 and AAS BSG 2, were added at a solid loading of 8 and 16% (w/v), respectively. The liquid fraction was then separated from the solid residue by centrifugation.

### 2.2. Determination of Chemical Composition of Raw and Pretreated Materials

Carbohydrate compositions of the biomass samples untreated and pretreated were determined according to the method of Davis [8]. The polysaccharides were degraded into their corresponding monomers by a two-step acid hydrolysis. The samples were mixed with a 72% v/v H_2_SO_4_ solution at a solid loading of 10% and incubated at 30°C for 1 h; then, the mixtures diluted to 4% (v/v) H_2_SO_4_ with distilled water were incubated for 1 hour at 120°C. After filtration through 0.45 *μ*m Teflon syringe filters (National Scientific, Lawrenceville, GA), the hydrolysis products were analyzed via high-performance liquid chromatography (HPLC) as described below. The acid insoluble lignin (Klason lignin) was determined, after total removal of the sugars by the weight of the dried residue.

### 2.3. Microorganisms and Cultivation Conditions for Screening in Synthetic Medium

The strains* Lactobacillus acidophilus* ATCC 53672,* L. acidophilus* ATCC 43121,* L. acidophilus* ATCC 4356,* L. lactis* INRA 18,* L. pentosus* NRRL B-227, and* L. plantarum* NRRL B-4496, all belonging to the strain collection of the Bioprocess and Biotechnology Division of the Department of “Engenharia de Bioprocessos e Biotecnologia” (Universidade Federal do Paraná, Brazil), were used in this study. Stock cultures were maintained at −20°C in 2 mL vials containing 25% (v/v) glycerol. The preinoculum was prepared by transferring 1 mL of stock culture cells to 25 mL tubes containing 10 mL of sterile Man, Rogosa, and Sharpe (MRS, Oxoid) culture medium with the following composition: 20 g/L glucose, 10 g/L peptone, 8 g/L meat extract, 4 g/L yeast extract, 2 g/L triammonium citrate, 2 g/L K_2_HPO_4_, 5 g/L CH_3_COONa·3H_2_O, 0.2 g/L MgSO_4_·7H_2_O, and 0.05 g/L MnSO_4_·4H_2_O. The tubes were statically incubated for 24 hours at 37°C. Fifty microliters of this culture was then transferred to a MRS agar Petri dish supplemented with 1 g/L Aniline Blue (Sigma-Aldrich). One single blue lactic bacterial colony was transferred into 15 mL sterile MRS broth medium contained within a 25 mL test tube. The tubes were statically incubated at 37°C for 48 hours. After incubation, 10 mL of this culture was transferred into a 250 mL Erlenmeyer flask containing 100 mL of MRS fermentation broth. The flasks were incubated at 37°C and 60 ×g for 96 hours during which time samples were withdrawn every 24 hours and subjected to analytical testing.

### 2.4. Inoculum and Fermentation Conditions for Cultivation in BSG Hydrolysate

The strain selected as the best producer of lactic acid in the primary screening was grown in 25 mL tubes containing 10 mL MRS broth. After 24 hours of static incubation at 37°C, 1 mL of this culture was transferred to a new tube with 10 mL MRS broth and incubated at the same conditions. Then, the cells were harvested by centrifugation (3.300 ×g for 15 minutes) and resuspended in distilled sterilized water. The glucose concentration of BSG hydrolysate was adjusted to 50 g/L with deionized water, while the pH was adjusted to a value of 6.0 by adding 5 M NaOH. The fermentation medium, with or without the addition of 1.25% (w/v) yeast extract, was then sterilized at 120°C for 15 minutes. Fermentations were performed in 250 Erlenmeyer flask containing 50 mL sterilized hydrolysate inoculated with 10% (v/v) of cell suspension. The flasks were incubated at 37°C and 60 ×g for 96 hours. All of the assays were performed in duplicate.

### 2.5. Analytical Methods

Samples taken during the fermentations at 24, 48, 72, and 96 hours were centrifuged at 12.800 g for 15 minutes. The resulting supernatant was used for measurement of glucose and lactic acid concentrations by high-performance liquid chromatography (HPLC). The HPLC analyses were performed in a Shimadzu LC-10-AD equipped with a C-RSA Integrator Chromatopac Chromatography Unit (Shimadzu) set to 210 nm (300 mm × 7.8 mm) column HPX-87-4 (Aminex) and a refractive index detector. The samples were diluted with deionized water, filtered through 0.2 *μ*m cellulose acetate filters (Sartorius Biolab Products), and then injected into the chromatograph. The conditions of chromatography involved a column temperature of 60°C, an injection volume of 50 *μ*L, and the elution with a mobile phase of 5 mM sulfuric acid at a flow rate of 0.6 mL/min.

The concentration of these compounds was calculated through calibration curves obtained using standard solutions. Also the sugars released from untreated and pretreated biomasses prepared as above described were estimated. Cell concentration was obtained by measuring the optical density (OD_600 nm_) in a spectrophotometer SP-2000 (Spectrum). Samples were diluted to a reading range of 0.05–0.5 OD units, and the OD values were correlated with the cell concentration (g/L) by means of a standard calibration curve previously established. The calibration curve for cell concentration and absorbance showed a linear relationship. One unit of optical density at 600 nm corresponded to approximately 0.6 g dry-cell weight/L.

### 2.6. Fermentative Parameters

The ratio of lactic acid produced to glucose consumed (g/g), the ratio of lactic acid (g/L) to fermentation time (h), the ratio of lactic acid to dry-cell weight (g/g), and the ratio of lactic acid yield per substrate consumed to the maximum theoretical value (1 g/g) were monitored during the fermentation of* Lactobacillus acidophilus* ATCC 43121.

## 3. Results and Discussion

### 3.1. Screening of Lactic Acid Bacteria for Lactic Acid Production in Synthetic Medium

**Figure 1:**
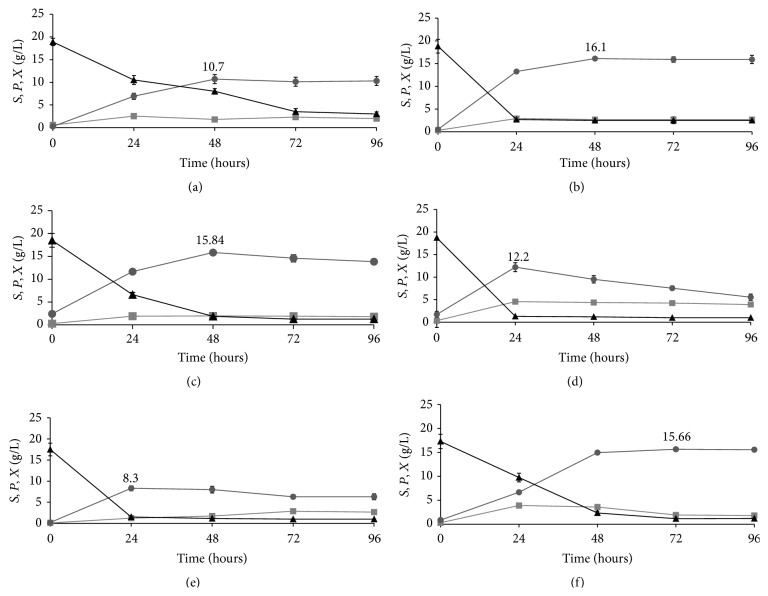
Time courses of glucose concentration (*S*, ∆), dry biomass concentration (*X*, □), and lactic acid concentration (*P*, o) during fermentation in synthetic medium of the* Lactobacillus* sp. strains: (a)* L. acidophilus* ATCC 53672, (b)* L. acidophilus* ATCC 43121, (c)* L. acidophilus* ATCC 4356, (d)* L. lactis* INRA 18, (e)* L. pentosus* NRRL b-227, and (f)* L. plantarum* NRRL b-4496. Data represent the mean of three independent experiments.

The lactic acid bacteria (LAB)* Lactobacillus acidophilus* ATCC 53672,* L. acidophilus* ATCC 43121,* L. acidophilus* ATCC 4356,* L. lactis* INRA 18,* L. pentosus* NRRL B-227, and* L. plantarum* NRRL B-4496 were compared for their lactic acid production ability in a synthetic medium. Since LAB have complex nutrient requirements, due to their limited ability to synthesize B-vitamins and amino acids [9], the Man, Rogosa, and Sharpe (MRS) broth containing minerals, B-vitamins, amino acids, fatty acids, purines, and pyrimidines was used as the fermentation medium. Time courses of dry biomass, glucose, and lactic acid concentration were monitored during batch fermentations of the six* Lactobacillus* strains in MRS broth (Figures 1(a)–1(f)).

The analyzed* Lactobacillus* strains showed significant differences in the fermentation performances, for example, cell growth and productivity. The maximum biomass varied from 1.9 ± 0.5 g/L for* L. acidophilus* ATCC 4356 (Figure 1(c)) to 4.56 ± 0.5 g/L for* L. lactis* INRA 18 (Figure 1(d)). Regarding the volumetric productivity, the values varied from a minimum of 0.22 g/L h and a maximum of 0.50 g/L h, reached by the strains* L. acidophilus* ATCC 53672 (Figure 1(a)) and* L. lactis* INRA 18 (Figure 1(d)), respectively. The maximum value of lactic acid production varied from 8.3 ± 0.6 g/L for* L. pentosus* NRRL B-227 (Figure 1(e)) to 16.1 ± 0.07 g/L for* L. acidophilus* ATCC 43121 (Figure 1(b)).* L. acidophilus* ATCC 43121 (Figure 1(b)),* L. acidophilus* ATCC 4356 (Figure 1(c)), and* L. plantarum* NRRL B-4496 (Figure 1(f)) showed the highest fermentation yield, with a maximum value of lactic acid production of 16.1 ± 0.07 g/L (after 48 hours), 15.84 ± 0.05 g/L (after 48 hours), and 15.66 g/L ± 0.04 (after 72 hours), respectively. The maximum *Y*_*P*/*S*_ value varied from a minimum of 0.52 g/g for the strain* L. pentosus* NRRL B-227 to a maximum of 0.99 g/g for* L. acidophilus* ATCC 43121, followed by the strains* L. acidophilus* ATCC 53672,* L. plantarum* NRRL B-4496,* L. acidophilus* ATCC 4356, and* L. lactis* INRA 18 with *Y*_*P*/*S*_ of 0.98 g/g, 0.96 g/g, 0.95 g/g, and 0.70 g/g, respectively.

*L. acidophilus* ATCC 43121, showing a volumetric productivity value of 0.34 g/L h and the highest lactic acid production, was selected for lactic acid production from the sugar mixture obtained by enzymatic hydrolysis of chemically pretreated BSG.

### 3.2. Chemical Pretreatments of Brewers' Spent Grains

BSGs from two different geographical areas were investigated as substrate for* L. acidophilus* ATCC 43121 growth and lactic acid production. The pretreatment of BSGs was performed by adopting two chemical methods. The biomass obtained from the brewery Bier Hoff Curitiba-PR Brazil, indicated as BSG 1, was subjected to an acid-alkaline treatment (AAT), whilst an alkaline pretreatment was conducted on the BSG from microbrewery Maneba Striano, Naples, Italy (BSG 2).

### 3.3. Acid-Alkaline Pretreatment

The acid-alkaline treatment (AAT) performed on the biomass obtained from the brewery Bier Hoff Curitiba-PR Brazil (BSG 1) involved two sequential steps accomplished through an acid impregnation followed by a biomass soaking in an alkaline solution. The procedure and operative conditions adopted were those tested by Mussatto and Roberto [5]. The BSG 1 was treated with 1.25% (v/v) H_2_SO_4_ to solubilize the hemicellulosic fraction. The initial removal of the hemicellulose shows the advantage of increasing the porosity of the material that facilitates the diffusion and impregnation of sodium hydroxide into the material sample, thus enhancing the soda pulping and the pulp uniformity. Moreover, since the hemicellulose is a valuable source of xylose, its recovery allows the sugar exploitation to produce value-added products such as xylitol [5] or ethanol [10]. After acid hydrolysis, a soda pulping pretreatment with 2% (v/v) NaOH was performed to liberate cellulose fibers from lignin [6].

Chemical analyses of the untreated and pretreated BSG 1 were performed to determine the macromolecular composition, and the results are reported in Table 1.

After pretreatment, the total solid biomass recovered was 7.89% (w/w). The hemicellulose was the main fraction removed (88.68% (w/w)), whilst a loss of 41.22% (w/w) and 84.54% (w/w) was obtained for lignin and other materials (ash, protein, and extractives), respectively. The cellulose content was 86.49% (w/w), corresponding to the main fraction of the pretreated BSG. However, if we considered that, after the pretreatment, the mass recovered from each 100 g of the original BSG was 7.89 g, the cellulose loss, calculated by the percentage of each fraction, was 52.65%.

### 3.4. Aqueous Ammonia Soaking Pretreatment

An alkaline pretreatment was conducted on the BSG 2 from microbrewery Maneba Striano, Naples, Italy, by soaking the lignocellulosic material in 5% ammonium hydroxide solution at 70°C for 22 h. As reported in literature, the alkali pretreatment is one of the most widely used methods to enhance the lignocellulosic biomass digestibility. One of the major advantages of this method is the limited hydrolysis of the (hemi)cellulosic fraction which prevents the production of sugar degradation products which inhibit the microbial fermentation, such as furfural [11]. The principal effect of the alkaline pretreatment is the lignin removal from the biomass through reactions of solvation and saponification of the intermolecular ester bonds among lignin, hemicellulose, and cellulose. Moreover, alkaline pretreatment causing the biomass swelling increases the lignocellulosic material porosity and reduces the polymerization degree and the crystallinity of the cellulose [12]. This in conjunction with the disruption of the lignin structure makes the cellulose more susceptible to enzymatic hydrolysis [11]. Soaking in aqueous ammonia solutions (AAS) is a common alkaline pretreatment that reduces lignin content and removes some hemicellulose while decrystallizing cellulose. At the mild conditions adopted in AAS, the level of end-groups dissolution, “peeling,” of the different polysaccharide chains is extremely limited thus avoiding carbohydrates loss and conversion of the lost sugars to compounds (e.g., furfural) with inhibitory effect on the fermentation process [13].

Chemical analyses of the untreated and AAS pretreated BSG 2 were performed to determine their macromolecular composition (Table 1). After pretreatment, the total solid biomass recovered was 60% (w/w). On the basis of the % composition of the material samples, a big fraction of lignin was removed (62.26%) whilst the cellulose and hemicellulose underwent an increase of 57.09 and 34.89%, respectively. With respect to the mass recovered from 100 g of the original BSG, calculated by the percentage of each fraction in 60 g of the pretreated material (total recovered mass after the pretreatment), it is evident that the highest losses were reported for the lignin fraction (77.3%) whilst only 19 and 5.8% of the hemicellulose and cellulose were removed, respectively. In agreement with the literature on the alkaline pretreatments, our results indicate that ammonium hydroxide pretreatment causes substantial lignin degradation whilst preserving most of the carbohydrates [14]. In particular, most of the cellulose was preserved probably due to the physical protection from lignin and hemicellulose. On the other hand, the heterostructures of xylan branched with short lateral chains contributed to the higher hemicelluloses hydrolysis [15].

### 3.5. Saccharification of the Pretreated BSGs by a Cocktail of Commercial Enzymes

The pretreated BSG samples were saccharified with commercial cellulase and *β*-glucosidase from Novozymes. The ratio of enzymes in the cocktail used for the saccharification was defined based on the composition of pretreated BSG samples reported in Table 1. The enzymatic saccharification of AAT BSG 1 and AAS BSG 2 generated hydrolysates containing 75 and 60 g/L of glucose, respectively. At the end of the reaction, 97 and 86.8% of efficiency of cellulose conversion into glucose were achieved for AAT BSG 1 and AAS BSG 2, respectively. These conversion yields are higher than that of 72% obtained by Mussatto et al. [16] on BSG pretreated with a similar acid-alkaline pretreatment. It can be observed that although the cellulose amounts (~90% w/w) in the biomasses employed in our saccharification experiments were comparable to those described by Mussatto et al. [16], the lignin ones were quite different. Thus, it is possible that the bigger lignin content (8.2% w/w) of the pretreated BSG utilized by Mussatto et al. [16] decreased the enzymes accessibility to the cellulose leading to the lower conversion yield.

### 3.6. Lactic Acid Production Using BSG Hydrolysates after Acid-Alkaline or Ammonia Soaking Pretreatment as Fermentation Media

**Figure 2:**
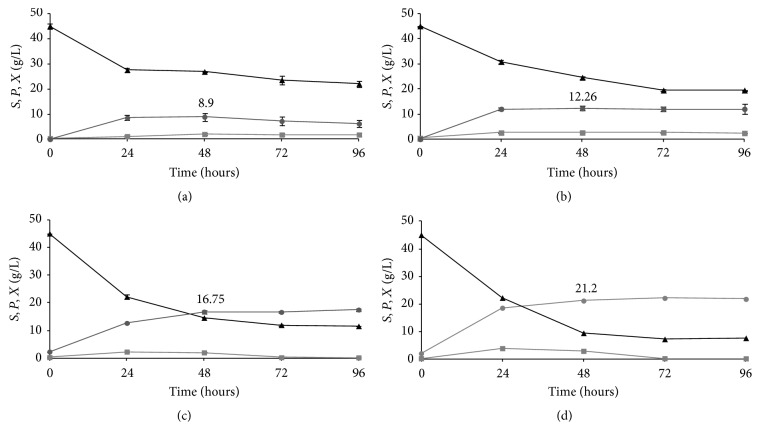
Time courses of glucose concentration (*S*, ∆), dry biomass concentration (*X*, □), and lactic acid concentration (*P*, o) during fermentation of* Lactobacillus acidophilus* ATCC 43121 in (a) acid-alkaline treated brewer's spent grain 1 hydrolysate, (b) acid-alkaline treated brewer's spent grain 1 hydrolysate + yeast extract, (c) aqueous ammonia soaking treated brewer's spent grain 2 hydrolysate, and (d) aqueous ammonia soaking treated brewer's spent grain 2 hydrolysate + yeast extract. All values are media of three replications.

The hydrolysates obtained from the saccharification of the differently pretreated BSGs were used to prepare the fermentation media for the lactic acid production by* Lactobacillus acidophilus* ATCC 43121, the strain previously selected among the analyzed LAB. The BSG hydrolysates, without adding any additional nutrients, and the BSG hydrolysates supplemented with the addition of 1.25% (w/v) yeast extract were adjusted to pH 6.0 and glucose concentration of 50 g/L by adding sterile water. It was observed that glucose consumption and lactic acid production occurred in all tested fermentation media (Figures 2(a)–2(d), Table 2).

The maximum value of lactic acid production was obtained from the AAS BSG 2 hydrolysate in the presence of yeast extract and it was 80% higher than that from the AAT BSG 1. Even in the absence of yeast extract, AAS BSG 2 hydrolysate allowed achieving a (around 96%) higher lactic acid production than the AAT BSG 1 hydrolysate. In any tested condition, the maximum production was observed after 48 hours.

The higher lactic acid production from AAS BSG 2 hydrolysate generates higher values of *Q*_*P*_ and *Y*_*P*/*X*_ than the AAT BSG 1 hydrolysate (Table 2).

It is worth noting that the glucose consumption increases in the AAS BSG 2 hydrolysate, resulting in being 46% higher than that measured in the AAT BSG 1 hydrolysate (Table 2), thus giving similar values of *Y*_*P*/*S*_ and fermentation efficiency for the two pretreatments. Moreover, in the AAT BSG 1 hydrolysate,* L. acidophilus* ATCC 43121 remained in a stationary phase until the end of fermentation whilst, in AAS BSG 2, the bacterium entered in early death phase at 72 h, which can be due to the poor residual glucose (Figures 2(a)–2(d)).

Using the hydrolysate from AAS BSG 2 allowed achieving higher biomass concentration than the AAT BSG 1, both in the presence and in the absence of yeast extract.

It is worthy of noting that the yield of lactic acid per substrate consumed (*Y*_*P*/*S*_) obtained using* L. acidophilus* ATCC 43121 grown in the BSG hydrolysate, without adding any additional element, was 0.48 g/g (from AAT BSG 1 hydrolysate) and 0.52 g/g (from AAS BSG 2 hydrolysate). This demonstrates that the BSG hydrolysate is an appropriate substrate for lactic acid production. Since the lactic acid is considered a relatively cheap product, the use of expensive carbon sources, such as glucose or starch, is not economical. Less expensive sources, like agroindustrial residues, are attractive alternatives.

The maximum lactic acid production levels shown in this study were higher than those obtained in the other few studies so far reported on the use of hydrolysates from agricultural residues as fermentation medium. The results of these studies are summarized in Table 3. In the best conditions, the values obtained in our studies for lactic acid production were higher than those reported by Jawad et al. [17], McCaskey et al. [18], and Mussatto et al. [19] and just a little lower than those obtained by Ali et al. [20]. As for the yields *Y*_*P*/*S*_, the maximum value obtained in our work was 14% lower than the maximum values previously reported [18, 19] indicating higher nutritional needs of* L. acidophilus* ATCC 43121 than the microbes adopted in the other studies. When the strain* L. acidophilus* ATCC 43121 was grown in the BSG hydrolysate supplemented with 1.25% (w/v) yeast extract, the yield of lactic acid increased from 0.48 g/g to 0.60 g/g (65% efficiency) for AAT BSG 1 hydrolysate and from 0.52 g/g to 0.61 g/g (65% efficiency) for AAS BSG 2 (Table 2).

Also the volumetric productivity (*Q*_*P*_) of lactic acid by* L. acidophilus* ATCC 43121 was higher in the BSG hydrolysate supplemented with yeast extract. Within 48 hours of fermentation, the cells produced a maximum value of 12.26 g/L of lactic acid from AAT BSG 1 hydrolysate and 22.16 g/L of lactic acid from the AAS BSG 2 hydrolysate with a volumetric productivity of 0.12 g/L h and 0.31 g/L h, respectively. These *Q*_*P*_ values are higher when compared with 0.09 g/L h and 0.18 g/L h produced in the AAT BSG 1 hydrolysate and the AAS BSG 2 without yeast extract addition, respectively (Table 2).

It is possible that the addition of yeast extract is necessary to reach an optimal C/N ratio for the lactic acid production by the* Lactobacillus* strain. These results are in agreement with those of many authors, showing the positive effect of BSG hydrolysate supplementation with additional nutrients, like yeast extract or vitamins, on lactic acid production. Yeast extract is the most commonly used nitrogen source, which provides the vitamin B complex content in addition to organic nitrogen to lactic acid bacteria.

Since the addition of yeast extract is disadvantageous from an economical point of view, more research will be needed to discover strains with lower nitrogen sources requests.

It was found that the maximum lactic acid productivity of the strain* L. casei* in a synthetic medium increased concomitantly with the initial yeast extract concentration. None of the other nitrogen sources tested gave lactic acid concentrations as high as that for yeast extract during 48 hours of fermentation [21].

Mussatto et al. [19] showed that the addition of 0.5% yeast extract enhanced the lactic acid production by* Lactobacillus delbrueckii*, reaching a concentration of ~9.0 g/L with a lactic acid yield (*Y*_*P*/*S*_) of 0.7 g/g. This is a higher value than that obtained from nonsupplemented hydrolysate (7.87 g/L). When fermented in BSG hydrolysate after AAS pretreatment without any nutrient supplementation, the* L. acidophilus* ATCC 43121 strain used in this study reached a lactic acid concentration of 17.49 g/L, a value higher than the one (~9.0 g/L) obtained by* L. delbrueckii* in the BSG hydrolysate supplemented with yeast extract [19]. Moreover, when the strain* L. acidophilus* ATCC 43121 was grown in the AAS BSG 2 hydrolysate supplemented with 1.25% yeast extract, a maximum lactic acid concentration of 22.16 g/L was reached. This concentration is 2.5-fold higher than that obtained by* Lactobacillus delbrueckii* in the presence of 0.5% yeast extract [19], which represents the main achievement of our study in comparison with the work performed on* Lactobacillus delbrueckii*.

## 4. Conclusions

The strain* Lactobacillus acidophilus* ATCC 43121 selected for its higher lactic acid production in a synthetic medium was analyzed for its ability to produce lactic acid from BSG hydrolysate. The results indicated that the produced hydrolysates from ammonia soaking treated BSG 2, provided by the microbrewery Maneba (Striano, Naples, Italy), and from the acid-alkaline treated BSG 1, provided by the brewery Bier Hoff Curitiba-PR (Brazil), are suitable substrates for the growth of the strain* L. acidophilus* LPB-04 and the production of lactic acid. Moreover, it was demonstrated that the bioconversion of glucose into lactic acid was positively affected by the presence of yeast extract. The maximum value of lactic acid production (22.16 g/L) was obtained from the AAS BSG 2 hydrolysate in the presence of yeast extract. The produced levels of lactic acid were comparable or higher in comparison with those obtained from hydrolysates of other agricultural residues. This result is in keeping with the many researches focused on the valorization of BSG in order to recover beneficial and valuable compounds, usable in nutrition or combustion field. Due to its composition and availability, in the last years, different methods to remove the water content, avoiding the BSG degradation [22–24], and to separate each component of it have been proposed [25, 26], taking different economical and ecofriendly advantages into account.

## Figures and Tables

**Table 1:** Chemical composition (% w/w) of brewer's spent grains in the original form and as a cellulose pulp recovered by acid-alkaline and aqueous ammonia soaking pretreatments.

Solid composition				
	Cellulose	Hemicellulose	Lignin	Others (ash, protein, and extractives)
	% (w/w)	% (w/w)	% (w/w)	% (w/w)
Untreated BSG 1^a^	14.42	34.21	3.93	47.43
Untreated BSG 2^b^	27.50	28.80	12.80	30.9
AAT^c^ BSG 1	86.49	3.87	2.31	7.33
AAS^d^ BSG 2	43.20	38.85	4.83	13.12

^a^BSG from the brewery Bier Hoff Curitiba-PR (Brazil).

^b^BSG from the microbrewery Maneba (Striano, Naples, Italy).

^c^BSG after acid-alkaline treatment (AAT).

^d^BSG after the aqueous ammonia soaking (AAS) treatment.

**Table 2:** Fermentative parameters of lactic acid production of *Lactobacillus acidophilus* ATCC 43121 by using hydrolysates of brewer's spent grain from the brewery Bier Hoff Curitiba-PR (Brazil) (BSG 1) after acid-alkaline treatment (AAT) and hydrolysates of brewer's spent grain from the microbrewery Maneba (Striano, Naples, Italy) (BSG 2) after aqueous ammonia soaking (AAS) treatment.

	Glucose consumption	Lactic acid	*Y*_*P*/*S*_^a^	*Y*_*P*/*X*_^b^	*Q*_*P*_^c^	*η* ^d^
	(g/L)	(g/L)	(g/g)	(g/g)	(g/L h)	(%)
AAT BSG 1 hydrolysate	18.20	8.90	0.48	4.45	0.09	48
AAT BSG 1 hydrolysate + yeast extract	20.50	12.26	0.60	4.69	0.12	60
AAS BSG 2 hydrolysate	33.50	17.49	0.52	7.64	0.18	52
AAS BSG 2 hydrolysate + yeast extract	37.40	22.16	0.61	5.61	0.31	61

^a^g-Lactic acid produced/g-glucose consumed.

^b^g-Lactic acid/g-dry-cell weight.

^c^(g/L)-Lactic acid/(h) fermentation time.

^d^ *Y*_*P*/*S*_/maximum theoretical value (1 g/g).

**Table 3:** Lactic acid production from agricultural residue hydrolysates.

Waste type	Waste pretreatment	Enzymatic hydrolysis	Fermentation conditions	Yield of glucose after enzymatic hydrolysis	Microorganism used in fermentation step	Production levels of lactic acid	Yield of lactic acid per glucose consumed	References
Brewer's spent grain	1.25% (v/v) sulfuric acid solution in a 1 : 8 g : g solid : liquid ratio, at 120°C for 17 minutes; 2% (v/v) sodium hydroxide solution in a 1 : 20 g : g solid : liquid ratio, at 120°C for 90 minutes	2.24% (v/v) cellulase (Novozymes) and 1% (v/v) *β*-glucosidase (Novozymes) using 8% (w/v) substrate, at 45°C and 120 rpm for 72 hours	Inoculum with 10% (v/v) of cell suspension, at 37°C and 60 ×g	75.0 g/L	*Lactobacillus acidophilus* ATCC 43121	8.9 g/L from AAT BSG 1 12.26 g/L from AAT BSG 1 + yeast extract 17.49 g/L from AAS BSG 2 22.16 g/L from AAS BSG 2 + yeast extract	0.48 g/g from AAT BSG 1 0.6 g/g from AAT BSG 1 + yeast extract 0.52 g/g from AAS BSG 2 0.61 g/g from AAS BSG 2 + yeast extract	This study

Mango peel	—	—	Coupling of the microbial hydrolysis and fermentation of the carbohydrate substrate into a single step at 35°C under static incubation conditions	—	Consortium of indigenous microorganisms of mango peels	17.48 g/L	—	[17]

Municipal solid waste (MSW)	2% (v/v) of H_2_SO_4_ (1 : 10 solid-to-liquid ratio) at 124°C for 15 minutes	—	Optimum conditions: initial pH 7.6, 1% v/v inoculum, and 5% (w/v) calcium carbonate buffer at 32°C	41.3 g/L	*Lactobacillus arabinosus* B-787, *L*. *arabinosus* B-788, *L*. *arabinosus* B-813, *L*. *arabinosus* B-531, *L*. *pentosus* B-277, *L*. *pentosus* B-473, *L*. *plantarum* USDA 422, and *L*. *xylosus* B-4449	~17–19 g/L excepted for *L*. *xylosus* B-4449 ~6 g/L	From 0.52 g/g to 0.73 g/g	[18]

Brewer's spent grain	1.25% (v/v) sulfuric acid solution in a 1 : 8 g : g solid : liquid ratio, at 120°C for 17 minutes; 2% (v/v) sodium hydroxide solution in a 1 : 20 g : g solid : liquid ratio, at 120°C for 90 minutes	Celluclast 1.5 L from *Trichoderma reesei* (Novozymes, Denmark)	Inoculated with an initial cell concentration of 1.0 g/L; statical incubation at 37°C	50.0 g/L	*Lactobacillus delbrueckii*	7.87 g/L using nonsupplemented BSG hydrolysate ~9 g/L using BSG hydrolysate supplemented with yeast extract	0.7 g/g	[19]

Corn cobs	5, 10, and 15% (v/v) of H_2_SO_4_ autoclaved at different temperatures for different intervals of time	—	Optimum conditions: 40°C, pH 5-6	4.0 (% w/w)	*Lactobacillus delbrueckii*	25.62 g/L	—	[20]

## References

[1] Liguori R., Amore A., Faraco V. (2013). Waste valorization by biotechnological conversion into added value products. *Applied Microbiology and Biotechnology*.

[2] Datta R., Tsai S. P., Bonsignore P., Moon S. H., Frank J. R. (1995). Technological and economic potential of poly(lactic acid) and lactic acid derivatives. *FEMS Microbiology Reviews*.

[3] Ghaffar T., Irshad M., Anwar Z. (2014). Recent trends in lactic acid biotechnology: a brief review on production to purification. *Journal of Radiation Research and Applied Sciences*.

[4] Mussatto S. I., Dragone G., Roberto I. C. (2006). Brewers' spent grain: generation, characteristics and potential applications. *Journal of Cereal Science*.

[5] Mussatto S. I., Roberto I. C. (2005). Acid hydrolysis and fermentation of brewer's spent grain to produce xylitol. *Journal of the Science of Food and Agriculture*.

[6] Mussatto S. I., Dragone G., Rocha G. J. M., Roberto I. C. (2006). Optimum operating conditions for brewer's spent grain soda pulping. *Carbohydrate Polymers*.

[7] Maurelli L., Ionata E., La Cara F., Morana A. (2013). Chestnut shell as unexploited source of fermentable sugars: effect of different pretreatment methods on enzymatic saccharification. *Applied Biochemistry and Biotechnology*.

[8] Davis M. W. (1998). A rapid modified method for compositional carbohydrate analysis of lignocellulosics by high pH anion-exchange chromatography with pulsed amperometric detection (HPAEC/PAD). *Journal of Wood Chemistry and Technology*.

[9] Morishita T., Deguchi Y., Yajima M., Sakurai T., Yura T. (1981). Multiple nutritional requirements of lactobacilli: genetic lesions affecting amino acid biosynthetic pathways. *Journal of Bacteriology*.

[10] Ferrari M. D., Neirotti E., Albornoz C., Saucedo E. (1992). Ethanol production from eucalyptus wood hemicellulose hydrolysate by *Pichia stipitis*. *Biotechnology and Bioengineering*.

[11] Singh J., Suhag M., Dhaka A. (2015). Augmented digestion of lignocellulose by steam explosion, acid and alkaline pretreatment methods: a review. *Carbohydrate Polymers*.

[12] Joshi B., Bhatt M. R., Sharma D., Joshi J., Malla R., Sreerama L. (2011). Lignocellulosic ethanol production: current practices and recent developments. *Biotechnology and Molecular Biology Review*.

[13] Taherzadeh M. J., Karimi K. (2008). Pretreatment of lignocellulosic wastes to improve ethanol and biogas production: a review. *International Journal of Molecular Sciences*.

[14] Gírio F. M., Fonseca C., Carvalheiro F., Duarte L. C., Marques S., Bogel-Łukasik R. (2010). Hemicelluloses for fuel ethanol: a review. *Bioresource Technology*.

[15] Hendriks A. T. W. M., Zeeman G. (2009). Pretreatments to enhance the digestibility of lignocellulosic biomass. *Bioresource Technology*.

[16] Mussatto S. I., Fernandes M., Dragone G., Mancilha I. M., Roberto I. C. (2007). Brewer's spent grain as raw material for lactic acid production by *Lactobacillus delbrueckii*. *Biotechnology Letters*.

[17] Jawad A. H., Alkarkhi A. F. M., Jason O. C., Easa A. M., Nik Norulaini N. A. (2013). Production of the lactic acid from mango peel waste—factorial experiment. *Journal of King Saud University—Science*.

[18] McCaskey T. A., Zhou S. D., Britt S. N., Strickland R. (1994). Bioconversion of municipal solid waste to lactic acid by *Lactobacillus* species. *Applied Biochemistry and Biotechnology*.

[19] Mussatto S. I., Fernandes M., Mancilha I. M., Roberto I. C. (2008). Effects of medium supplementation and pH control on lactic acid production from Brewer's spent grain. *Biochemical Engineering Journal*.

[20] Ali Z., Anjum F. M., Zahoor T. (2009). Production of lactic acid from corn cobs hydrolysate through fermentation by *Lactobaccillus delbrukii*. *African Journal of Biotechnology*.

[21] Yoo I.-K., Chang H. N., Lee E. G., Chang Y. K., Moon S.-H. (1997). Effect of B vitamin supplementation on lactic acid production by *Lactobacillus casei*. *Journal of Fermentation and Bioengineering*.

[22] Carvalheiro F., Esteves M. P., Parajó J. C., Pereira H., Gírio F. M. (2004). Production of oligosaccharides by autohydrolysis of brewery's spent grain. *Bioresource Technology*.

[23] Mandalari G., Faulds C. B., Sancho A. I. (2005). Fractionation and characterisation of arabinoxylans from brewers' spent grain and wheat bran. *Journal of Cereal Science*.

[24] Tang D.-S., Yin G.-M., He Y.-Z. (2009). Recovery of protein from brewer's spent grain by ultrafiltration. *Biochemical Engineering Journal*.

[25] El-Shafey E. I., Gameiro M. L. F., Correia P. F. M., de Carvalho J. M. R. (2004). Dewatering of brewer's spent grain using a membrane filter press: a pilot plant study. *Separation Science and Technology*.

[26] Fernández M. P., Rodriguez J. F., García M. T., de Lucas A., Gracia I. (2008). Application of supercritical fluid extraction to brewer's spent grain management. *Industrial and Engineering Chemistry Research*.

